# A novel mitochondrial DnaJ/Hsp40 family protein BIL2 promotes plant growth and resistance against environmental stress in brassinosteroid signaling

**DOI:** 10.1007/s00425-013-1859-3

**Published:** 2013-03-15

**Authors:** Davaapurev Bekh-Ochir, Setsuko Shimada, Ayumi Yamagami, Satomi Kanda, Kenji Ogawa, Miki Nakazawa, Minami Matsui, Masaaki Sakuta, Hiroyuki Osada, Tadao Asami, Takeshi Nakano

**Affiliations:** 1 RIKEN Advanced Science Institute, 2-1 Hirosawa, Saitama Wako, 351-0198 Japan; 2 RIKEN Genome Science Center, Tsurumi, Kanagawa Yokohama, 230-0045 Japan; 3 RIKEN Plant Science Center, Tsurumi, Kanagawa Yokohama, 230-0045 Japan; 4 Department of Biology, Ochanomizu University, Tokyo, 112-8610 Japan; 5 JST, CREST, 4-1-8 Honcho, Saitama Kawaguchi, 332-0012 Japan; 6 Department of Applied Biological Chemistry, Graduate School of Agricultural and Life Sciences, The University of Tokyo, Yayoi, Bunkyo-ku, Tokyo, 113-8657 Japan

**Keywords:** ATPase, Brassinosteroid, Cell elongation, Chemical biology, DnaJ/Hsp40, Mitochondria

## Abstract

Plant steroid hormones, brassinosteroids, are essential for growth, development and responses to environmental stresses in plants. Although BR signaling proteins are localized in many organelles, i.e., the plasma membrane, nuclei, endoplasmic reticulum and vacuole, the details regarding the BR signaling pathway from perception at the cellular membrane receptor BRASSINOSTEROID INSENSITIVE 1 (BRI1) to nuclear events include several steps. Brz (Brz220) is a specific inhibitor of BR biosynthesis. In this study, we used Brz-mediated chemical genetics to identify *Brz*-*insensitive*-*long hypocotyls 2*-*1D* (*bil2*-*1D*). The *BIL2* gene encodes a mitochondrial-localized DnaJ/Heat shock protein 40 (DnaJ/Hsp40) family, which is involved in protein folding. *BIL2*-overexpression plants (*BIL2*-*OX*) showed cell elongation under Brz treatment, increasing the growth of plant inflorescence and roots, the regulation of BR-responsive gene expression and suppression against the dwarfed *BRI1*-deficient mutant. *BIL2*-*OX* also showed resistance against the mitochondrial ATPase inhibitor oligomycin and higher levels of exogenous ATP compared with wild-type plants. BIL2 participates in resistance against salinity stress and strong light stress. Our results indicate that BIL2 induces cell elongation during BR signaling through the promotion of ATP synthesis in mitochondria.

**Electronic supplementary material:**

The online version of this article (doi:10.1007/s00425-013-1859-3) contains supplementary material, which is available to authorized users.

## Introduction

Brassinosteroids (BRs) are plant steroid hormones that regulate various processes including plant growth and responses to environmental stress, among others. Mutants deficient in BR biosynthesis or signal transduction exhibit phenotypes such as de-etiolated hypocotyls and opened cotyledons in the dark and dwarfism with shortened leaves and stems in the light (Li et al. 1996; Li and Chory 1997). The activation of BR biosynthesis and signal transduction promotes hypocotyl and stem elongation and outward leaf curling. These results showed that BR is necessary for the positive cell elongation and growth of the plant (Belkhadir and Chory 2006).

BR signaling factors are localized in various parts and organelles in the plant cell. BRs are perceived through a plasma membrane localized receptor BRASSINOSTEROID INSENSITIVE 1 (BRI1), a leucine-rich repeat receptor-like serine/threonine kinase that functions in cell elongation (Kinoshita et al. 2005). BRI1 works with the negative regulator BRI1 KINASE INHIBITOR 1 (BKI1) (Wang and Chory 2006) and the positive regulator BRI1-ASSOCIATED RECEPTOR KINASE 1 (BAK1) (Li et al. 2002; Nam and Li 2002), which are anchored to the plasma membrane. BR signals received through these factors on the cell surface are transduced to *bri1*-*5* SUPPRESSOR 1 (BSU1), which is a positive phosphatase (Mora-Garcia et al. 2004) and BRASSINOSTREROID INSENSITIVE 2 (BIN2), which is a negative kinase similar to the glycogen synthase kinase 3-like kinase (Li and Nam 2002) in the cytosol. BIN2 kinase and BSU1 dephosphorylation activities regulate the phosphorylation of BRASSINOZOLE RESISTANT 1/Brz-INSENSITIVE-LONG HYPOCOTYLS 1 (BZR1/BIL1) (Wang et al. 2002; He et al. 2005; Asami et al. 2005) and *bri1*-EMS-SUPPRESSOR 1 (BES1) (Yin et al. 2002, 2005), which regulate gene expression in the nucleus. Although BR signal transduction from the plasma membrane to the cytosol and nuclei has been described, the BR signaling factors in other organelles have not been clarified.

ATP is well known as an energy source for the growth and life cycle in organisms from plant to animal. Plasma membrane P-ATPase in plants is activated by BRI1 kinase to induce cell wall expansion (Caesar et al. 2011). Reduced glutathione and dithiothreitol inhibited plant hypocotyl elongation; however, hypocotyl elongation could be recovered after treatment with ATP. NADPH oxidase activity and the endogenous production of nitric oxide positively affected hypocotyl elongation with the help of ATP treatment (Tonon et al. 2010). Translocase of the inner membrane 50 (TIM50) and translocase of the inner membrane 21 (TIM21) are mitochondrial proteins, and knock-out mutants of these genes showed a reduction of intracellular ATP levels and short hypocotyls in the dark (Hamasaki et al. 2012; Kumar et al. 2012). These results suggested that ATP plays an important role in plant growth, but a regulatory mechanism between the other plant growth regulator plant hormone and ATP itself is still unknown.

Brz is a specific inhibitor of BR biosynthesis. Brz treatment caused BR-deficient mutant like phenotype onto wild-type plant (Asami et al. 2000). Here, we have isolated and characterized an *Arabidopsis* mutant, *Brz*-*insensitive*-*long hypocotyls 2*-*1D* (*bil2*-*1D*) using activated tagging mutant lines by Brz. The *bil2*-*1D* mutant exhibited positively regulated growth in the hypocotyl, branch and root. *bil2*-*1D* responsible gene encodes a novel DnaJ/Hsp40 family protein that is localized in the mitochondria. In this manuscript, we attempted to identify the mitochondrial protein involved in plant growth under BR signal transduction.

## Materials and methods

### Plant materials, growth conditions and stress treatments

*Arabidopsis thaliana* ecotype Columbia (Col-0) was used as the wild-type plant. Seeds were germinated on medium containing 1/2 Murashige and Skoog (MS) medium (Duchefa, Haarlem, The Netherlands) and 0.8 % phytoagar (Duchefa, Haarlem, The Netherlands) with 1.5 % sucrose and were subsequently transferred to soil. The plants were grown at 22 °C under white light (a 16 h light/8 h dark cycle for long-day conditions). For the genetic analysis of double crossing, the *bri1*-*5* (Wassilewskija[Ws-2]) mutant was used. Seeds of WT and *bri1*-*5* mutant were obtained from ABRC (Arabidopsis Biological Resource Center, Ohio University, Columbus, OH, USA). For the ATP experiment, adenosine 5′-triphosphate disodium salt hydrate (ATP; Sigma-Aldrich, St. Louis, USA) at concentrations of 125 and 250 μM was added to 1/2 MS medium. The ATPase inhibitor oligomycin (Calbiochem, Darmstadt, Germany) was used at concentrations of 25 and 50 μM. The seeds were germinated in darkness for 7 days at 22 °C. To induce salt stress, the seeds were germinated on 1/2 MS plates containing 0 and 125 mM of NaCl for 25 days. For the strong light stress analysis, the seeds were germinated on 1/2 MS medium under strong light (486.2 μmol m^−2^ s^−1^) for 25 days. Control plants were germinated in normal light (92.27 μmol m^−2^ s^−1^).

### Screening for *bil2*-*1D* mutants

Approximately 10,000 of the RIKEN GSC *Arabidopsis* activation tagging lines (Nakazawa et al. 2003) were screened on 1/2 MS medium containing 3 μM Brz (Asami et al. 2000). After growth for 7 days in the dark, seedlings with hypocotyls longer than the controls were identified and transferred to the soil. TAIL-PCR was used to amplify the flanking genomic sequences of the T-DNA of pPCVICE4HPT, as previously described. Total RNA was extracted from the dark-grown 3-day-old seedlings of wild type and *bil2*-*1D* plants using an RNeasy Plant Mini Kit (Qiagen, Hilden, Germany). First-strand cDNA was synthesized with PrimeScript (Takara, Kyoto, Japan), and used in quantitative real-time PCR (qRT-PCR). The qRT-PCR analysis was performed according to the instructions provided for the Thermal Cycler Dice (Takara) using a SYBR Premix ExTaq system (Takara). The following gene-specific primers were used for qRT-PCR analysis: for *BIL2*, 5′-TGAGTCCCTCAGGTCCCTTA-3′ and 5′-GCCGCCTCCTTGGTAGAG-3′; and for the constitutively expressed control gene *ACT2*, 5′-CGCCATCCAAGCTGTTCTC-3′ and 5′-TCACGTCCAGCAAGGTCAAG-3′.

### Generating transgenic plants

In the recapitulation of the *bil2*-*1D* phenotype, *BIL2* cDNA was amplified from *Arabidopsis* Col-0 cDNA with primers for *BIL2*-Forward 5′-CACCATGAACGCCGCCATTAGAGC-3′, and *BIL2*-Reverse 5′-TTAGTTCACAGATAGCTTTTCACA-3′ and cloned into pENTR/D-TOPO and subsequently cloned into the binary vector pGWB2 (Invitrogen; Nakagawa et al. 2007) containing a CaMV 35S promoter using a Gateway strategy. To generate RNAi constructs of *BIL2*, pENTR-*BIL2* was subsequently cloned into the binary vector pGWB80 using a Gateway strategy.

For subcellular localization, the *BIL2*-*GFP* construct was generated. A 3-kb fragment, including the promoter region and open reading frame (ORF) of *BIL2*, was amplified from *Arabidopsis* Col-0 cDNA using primers for the *BIL2 promoter: BIL2*-*GFP*-Forward 5′-CACCTTGGAGAGAGATCAAAGAGGAACAATC-3′ and *BIL2*-*GFP*-Reverse 5′-GTTCACAGATAGCTTTTCACATAAAGA-3′. The PCR fragment was cloned into pENTR/D-TOPO and subsequently cloned into the binary vector pGWB5 using a Gateway strategy (Invitrogen, Carlsbad, CA, USA).

For the *BIL2* promoter and GUS fusion construct, a 1.1-kb fragment, including the first exon and promoter region, was amplified from *Arabidopsis* Col-0 genomic DNA using primers for *BIL2*-*GUS*-Forward 5′-CACCTTGGAGAGAGATCAAAGAGG-3′ and *BIL2*-*GUS*-Reverse 5′-CGATTCCCAGGAAGTGCGAC-3′ and cloned into pENTR/D-TOPO and subsequently cloned into the binary vector pGWB3 using a Gateway strategy (Invitrogen).

The resulting p35S-*BIL2,* *BIL2 promoter:BIL2*-*GFP* and *BIL2* promoter and GUS fusion constructs were transformed into *Col*-*0* using the floral dipping method. The transgenic plants were screened on 1/2 MS medium containing 25 mg/l of kanamycin.

### Quantitative real-time PCR

Total RNA was extracted using an RNeasy Plant Mini Kit (Qiagen) from light-grown, wild-type 24- (*BIL2*-*OX*) and 7-day-old (*BIL2*-*RNAi*) seedlings; dark-grown 5-day-old (genes flanking the T-DNA insertion site) seedlings of wild-type and *bil2*-*1D* plants; and wild-type and *bil2*-*1D* plants transformed with *BIL2*-*OX* or *BIL2*-*RNAi*. The first-strand cDNA was synthesized using PrimeScript (Takara) and was used in quantitative real-time PCR (qRT-PCR). The qRT-PCR was performed according to the instructions provided for the Thermal Cycler Dice (Takara) using the SYBR Premix ExTaq system (Takara).

The following primers were used: *TCH4*-Forward 5′-CGAGTCTTGGAACGCTGAT-3′ and *TCH4*-Reverse 5′-CTTCTTGTTGAAAGCCACGG-3′; *CPD*-Forward 5′-CACTTCAAAGATGCTCGCACTT and *CPD*-Reverse 5′-CAGCTCGTAACCGGGACATAG-3′; At2g42020-Forward 5′-GCTTTGGCCGAGTGGTTAAG-3′ and At2g42020-Reverse 5′-AACTCTCGCGGGGAAAC-3′; At2g42030-Forward 5′-AGCCAGCGACGAATTA-3′ and At2g42030-Reverse 5′-CGTCTGAAACGTGACC-3′; At2g42040-Forward 5′-CGTGCATGTGATAAGTAGG-3′ and At2g42040-Reverse 5′-CACACACCCAATACATAGAG-3′; At2g42060-Forward 5′-CTCTGAATCCGAAACC-3′ and At2g42060-Reverse 5′-CAGCGTGAGAAGGATG-3′; At2g42070-Forward 5′-TTCAGATATTCGCGCCTTTA-3′ and At2g42070-Reverse 5′-GGAAGAACAAAGCCAATCCA-3′.

### GUS staining

For histochemical detection of GUS expression 2-, 3-, 11- and 28-day-old seedlings of *promoter:GUS:BIL2* transgenic plants were used. The samples were stained at 37 °C overnight in GUS staining solution as previously described (Ito and Fukuda 2002). To test the induction of GUS expression, 2- and 3-day-old transgenic seedlings were treated with brassinolide (BL) and Brz for 3 h.

### Subcellular localization analysis by fluorescence microscopy

The roots of 5-day-old *BIL2*-*GFP* transgenic seedlings were harvested into a freshly prepared staining solution of 500 nM CM-H2XRos (MitoTracker Red; Invitrogen) for 15 min at room temperature. After staining, the seedlings were washed three times in 1/2 MS medium for approximately 10 min (Hedtke et al. 1999).

XylT (beta-1,2-xylosyltransferase)::RFP and HDEL (His-Asp-Glu-Leu)::RFP were generated by modifying XylT-GFP and HDEL-GFP and cloning into pGWB (Shoda et al. RIKEN Advanced Science Institute, Saitama, Japan, unpublished data). The *BIL2*-*GFP* transformant was generated as previously described. These plant organelle marker-RFP constructs were transformed into *BIL2*-*GFP* plants using floral dipping. Images of *BIL2*-*GFP* and Mitotracker fluorescence were captured using a LSM 700 laser scanning microscope (Zeiss, Oberkochen, Germany).

### ATP measurement

Five-day-old dark mature seedlings were used. The samples were frozen in liquid N_2_ after harvest and stored at −80 °C. The samples were immersed in sterile water and boiled for 15 min at 98 °C to destroy any ATPases (Yang et al. 2002). Total ATP content in the supernatant was determined using a luciferase-based assay (Kikkoman, Tokyo, Japan), and the luminescence was measured in a Spectra MaX luminometer (Molecular Devices, Sunnyvale, CA, USA). The ATP content was correlated with luminescence by comparison with the ATP standard provided in the kit.

## Results

### Isolation and characterization of the *bil2*-*1D* mutant

Brz is a triazole-type compound that directly binds to the cytochrome P450 steroid C-22 hydroxylase encoded by the *DWARF4* (*DWF4*) gene and specifically inhibits BR biosynthesis. Brz treatment reduces the BR content in plant and causes phenotypes with de-etiolation and dwarfism similar to BR-deficient mutants (Asami et al. 2000, 2001). Mutants that are insensitive to Brz can be activated in BR signaling and biosynthesis; therefore, we screened the *brz*-*insensitive*-*long hypocotyl* (*bil*) mutant in *Arabidopsis*. We have isolated the *bzr1*/*bil1* mutant from EMS-mutation lines, and identified BZR1/BIL1 with a bHLH transcription factor that acts as a positive regulator in BR signaling (Wang et al. 2002; Asami et al. 2005; He et al. 2005).

**Fig. 1:**
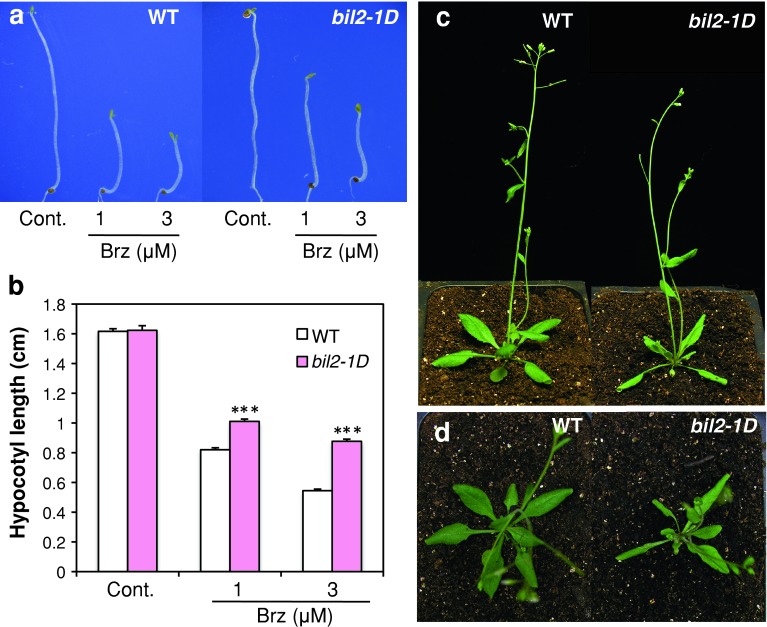
Phenotype and characterization of *bil2*-*1D*. *bil2*-*1D* mutant showed Brz resistance (**a**, **b**). **a** Hypocotyl elongation of wild-type (WT) and *bil2*-*1D* seedlings grown on medium containing 0, 1, and 3 μM Brz in the dark for 7 days. **b** Hypocotyl length of wild-type (WT) and *bil2*-*1D* seedlings grown on medium containing 0, 1 and 3 μM Brz in the dark for 7 days. The results are presented as the mean ± SE (*n* > 30 seedlings). *Triple asterisk* indicates significant differences relative to the control at *P* < 0.001 based on the Student’s *t* test. **c**, **d** Phenotype of wild type and *bil2*-*1D* seedlings grown in soil under long-day conditions (16 h light, 8 h dark) for 30 days. Side view (**c**) and top view (**d**)

In the dark, wild-type plants are typically etiolated with an elongated hypocotyl and a closed cotyledon. Treatment with Brz inhibits the elongation of hypocotyls and enhances the opening of the cotyledon in the wild-type plant, termed as de-etiolation in the dark. Under these growth conditions, we screened 10,000 *Arabidopsis* activation tagged lines and isolated a semi-dominant mutant, *Brz*-*insensitive*-*long* *hypocotyls 2*-*1D* (*bil2*-*1D*). *bil2*-*1D* mutant seedlings showed longer hypocotyls and closed cotyledons when grown in the dark on a medium containing Brz (Fig. 1a, b). In the dark without Brz, the hypocotyls of *bil2*-*1D* plants were elongated normally and were similar in length to the wild type.

Light-grown *bil2*-*1D* mutants showed long petioles and outward-curling leaves similar to the overexpressed plants of the BR receptor, BRI1 (Wang et al. 2001) (Fig. 1c, d). These phenotypes suggested that *bil2* mutants enhanced BR signaling.

### Identification and characterization of the *BIL2* gene

**Fig. 2:**
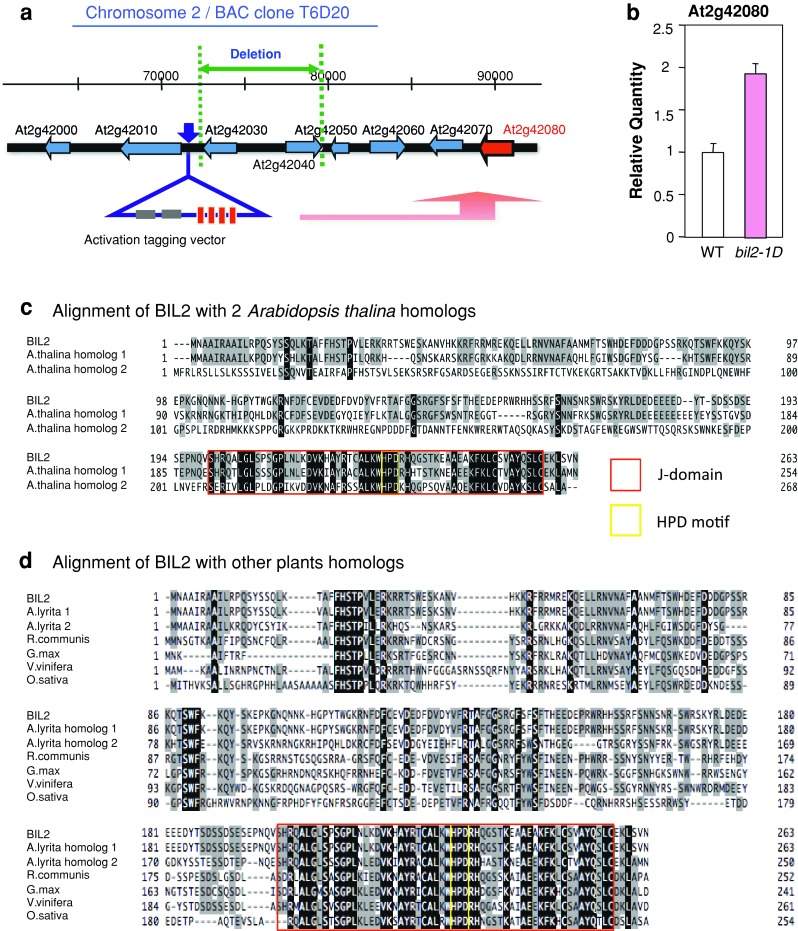
Novel gene is candidate of *bil2*-*1D* mutant. **a** T-DNA insertion site in *bil2*-*1D* is indicated with *blue*, deletion site with green and the *BIL2* gene with a *red arrow*. **b** Quantitative real-time PCR analysis of At2g42080 mRNA expression in dark-grown, 3-day-old wild-type (WT) and *bil2*-*1D* seedlings. The value was normalized against the expression of the *ACTIN2* gene. The *error bars* indicate standard deviation (*n* = 3). **c** Alignment of BIL2 (NP_181738) and its homologs in *Arabidopsis thaliana*, At3g58020 (NP_191361) and At2g18465 (NP_849977). **d** Alignment of BIL2 (NP_181738) and its homologs in other plants: *Arabidopsis lyrata* 1 (XP_002881820), *Arabidopsis lyrata* 2 (XP_002876451), castor bean *Ricinus communis* (XP_002530712), soybean *Glycine max* (XP_003519035), grape *Vitis vinifera* (XP_002279725), and rice *Oryza sativa* (NP_001059604). The *red box* indicates the J domain, and *yellow box* indicates the HPD motif, which is important for the J domain

The co-segregation of the Brz-insensitive phenotype with the selection marker and TAIL-PCR indicated a T-DNA insertion in an intergenic region at the end of chromosome II in *bil2*-*1D* (Fig. 2a). The At2g42080 gene, located approximately 14.3 kbp downstream of the T-DNA insertion, was overexpressed (Fig. 2b). From T-DNA insertion to the At2g42080 gene, a 7.3-kbp gene was deleted (Fig. 2a). The knock-out mutant for At2g42030 (SALK_061281.28.80.x) and At2g42040 (SALK_007993) demonstrated the expression of each mRNA (Suppl. Fig. S1) and the same phenotype as the wild-type plant (data not shown). The increased expression of the At2g42060 and At2g42070 genes was also identified in *bil2*-*1D* compared with the wild type (Suppl. Fig. S1). The transformation and overexpression of genes for At2g42060 and At2g42070 in the wild-type plant showed the same hypocotyl and leaf phenotype as the wild-type plant (data not shown). These results suggested that the *bil2*-*1D* mutation is due to the mRNA overexpression of At2g42080 and the gene is named as *BIL2*.

*BIL2* encodes a novel protein, but is categorized in DnaJ/Heat shock protein 40 (Hsp 40) family, which function as molecular chaperones (Rajan and D’Silva 2009). Ordinary DnaJ/Hsp40 gene family function has chaperone activity to repair the misfolding of nascent polypeptides and protein aggregation during stress (Hartl 1996). DnaJ/Hsp40 contains a J domain, which is essential for interaction with DnaK/Hsp70, and this domain is conserved in BIL2 (Fig. 2c, d). The J domain contains a highly conserved histidine, proline and aspartate (HPD) motif, which is critical for the function of these proteins (Cheetham and Caplan 1998). A BLAST search revealed that two homologous genes of *BIL2* were present in *Arabidopsis thaliana*: At3g58020 and At2g18465 (Fig. 2c) and some homologous genes in *Arabidopsis lyrata*, castor bean (*Ricinus communis*), soybean (*Glycine max*), grape (*Vitis vinifera*) and rice (*Oryza sativa*) (Fig. 2d).

### The *BIL2* gene promotes plant growth

**Fig. 3:**
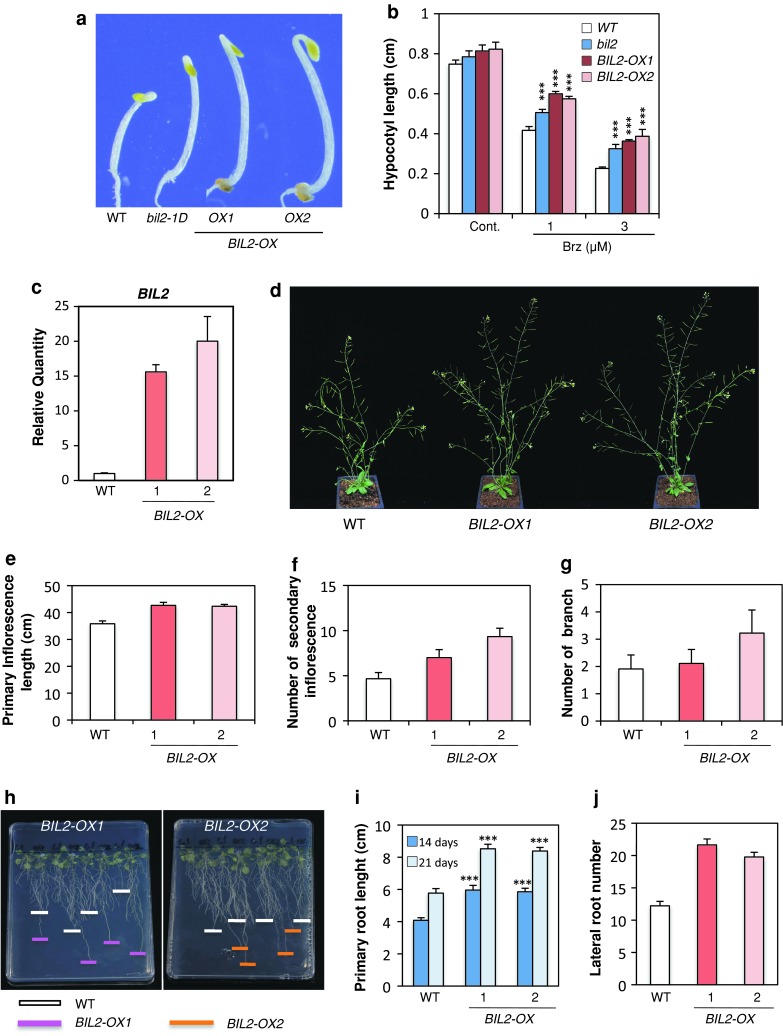
*BIL2*-*OX* increased the growth of the hypocotyl, inflorescence and roots. **a** Hypocotyl elongation of wild-type (WT), *bil2*-*1D*, *BIL2*-*OX1* and *BIL2*-*OX2* seedlings grown on medium containing 3 μM Brz in the dark for 4 days. **b** Hypocotyl length of wild type (WT), *bil2*-*1D*, *BIL2*-*OX1* and *BIL2*-*OX2* seedlings grown on medium containing 0, 1 and 3 μM Brz in the dark for 4 days. The results are presented as the mean ± SE (*n* > 30 seedlings). **c** Real-time PCR analysis of the *BIL2* gene expression in the wild-type (WT), *BIL2*-*OX1* and *BIL2*-*OX2* seedlings grown in the light for 24 days. The value was normalized against the expression of the *ACTIN2* gene. The *error bars* indicate standard deviation (*n* = 3). **d** Phenotype of wild-type (WT), *BIL2*-*OX1* and *BIL2*-*OX2* seedlings grown in soil under long-day conditions (16 h light, 8 h dark) for 40 days. **e**–**g** Measurements indicating primary inflorescence length (**e**), secondary inflorescence number (**f**) and branch number (**g**) of wild-type (WT), *BIL2*-*OX1* and *BIL2*-*OX2* seedlings grown on soil for 40 days. The results are presented as the mean ± SE (*n* > 15 plants). **h** Root elongation of wild-type (WT), *BIL2*-*OX1* and *BIL2*-*OX2* seedlings grown on 1/2MS medium light 14 days. Wild-type (WT) is shown in *white*, *BIL2*-*OX1* in *pink* and *BIL2*-*OX2* in *orange*. **i** Primary root length of wild-type (WT), *BIL2*-*OX1* and *BIL2*-*OX2* grown on 1/2 MS medium in light for 14 and 21 days. **j** Lateral root number for seedlings grown in light for 14 days. *Triple asterisk* indicates significant differences relative to the control at *P* < 0.001 based on the Student’s *t* test

To confirm that the overexpression of At2g42080 caused the *bil2*-*1D* phenotype, the At2g42080 coding region was placed immediately downstream of the CaMV 35S promoter and transformed into wild-type *Arabidopsis*. The obtained *BIL2* over-expresser (*BIL2*-*OX*) showed long hypocotyls and closed cotyledons when grown in the dark on medium containing Brz (Fig. 3a, b).

To analyze the role of *BIL2* for plant growth, *BIL2*-*OX* and *BIL2*-*RNAi* transformants were generated and observed in detail. *bil2*-*1D* contains two gene deletions (At2g42030 and At2g42040).

Although the deletion of the two genes did not affect the *bil2*-*1D* phenotype (Suppl. Fig. S1), the potentiation effect of the gene deletion must be avoided. Because the *BIL2* over-expresser exhibits BIL2 function, we used these transformants for further analysis.

In plants grown in soil under light, the primary inflorescence length of *BIL2*-*OX* expressing *BIL2* mRNA was longer than that of the wild-type plants (Fig. 3c–e). Although the number of primary inflorescences was similar between *BIL2*-*OX* and wild-type plants (data not shown), the number of secondary inflorescences and branches in *BIL2*-*OX* were increased compared with the wild-type plant (Fig. 3f, g). *BIL2*-*OX* showed longer roots and increased lateral root numbers compared with the wild-type plant (Fig. 3h–j).

**Fig. 4:**
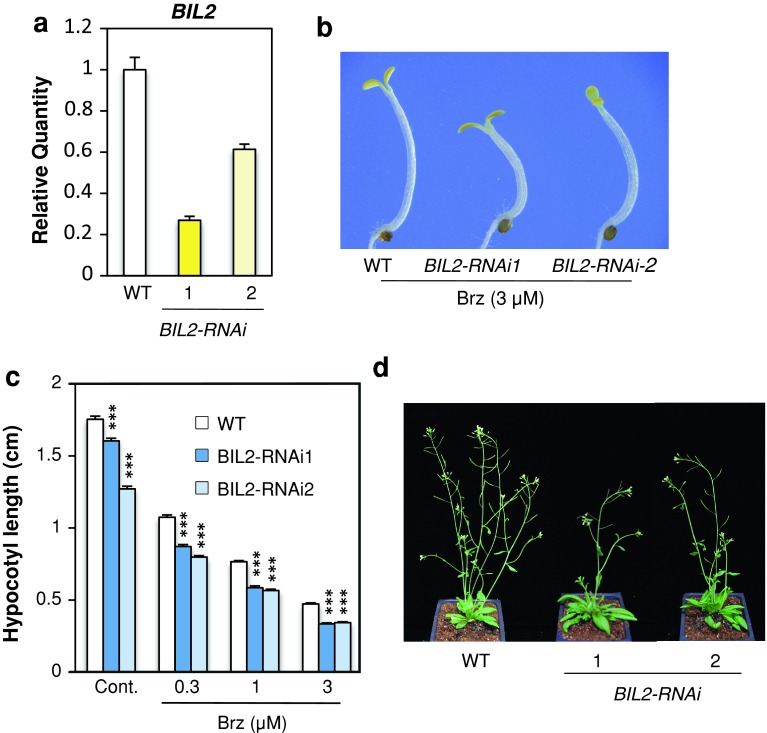
The *BIL2*-*RNAi* mutant showed short hypocotyls and short inflorescence. **a** Real-time PCR analysis of the *BIL2* gene expression in the wild-type (WT), *BIL2*-*RNAi1* and *BIL2*-*RNAi2* seedlings grown in the light for 7 days. The value was normalized against the expression of the *ACTIN2* gene. The *error bars* indicate standard deviation (*n* = 3). **b** Hypocotyl elongation of wild type (WT) and *BIL2*-*RNAi1* and *BIL2*-*RNAi2* seedlings grown on medium containing 3 μM Brz in the dark for 7 days. **c** Hypocotyl length of wild-type (WT), *BIL2*-*RNAi1* and *BIL2*-*RNAi2* grown on medium containing 0, 0.3, 1 and 3 μM Brz in the dark for 7 days. The results are presented as the mean ± SE (*n* > 30 seedlings). *Triple asterisk* indicates significant differences relative to the control at *P* < 0.001 based on the Student’s *t* test. **d** Phenotype of wild type (WT), *BIL2*-*RNAi1* and *BIL2*-*RNAi2* seedlings grown in soil under long-day conditions (16 h light, 8 h dark) for 40 days

In contrast, *BIL2*-RNA interference vector was constructed downstream of the CaMV 35S promoter and transformed into wild-type *Arabidopsis* (*BIL2*-*RNAi*). Two *BIL2*-*RNAi* lines showed that *BIL2* mRNA was decreased compared with the wild-type plant (Fig. 4a), showing shorter hypocotyls than wild-type plants when grown in the dark with and without Brz. The shortened hypocotyl of the *BIL2*-*RNAi* plants showed a dose-dependent response to the Brz concentration (Fig. 4b, c). Compared with the wild-type plants, the *BIL2*-*RNAi* lines did not have decreased homologous genes of *BIL2* in *Arabidopsis thaliana* (Suppl. Fig. S2). Although we could not obtain and analyze the *BIL2* gene knock-out mutant, the *BIL2*-*RNAi* phenotype suggested an effect of the *BIL2* single knockdown. Light-grown *BIL2*-*RNAi* transformants showed shorter inflorescence and a tendency of decreased branches compared with wild-type plants (Fig. 4d). These results suggest that BIL2 plays an important role in positive plant growth.

### The BR-responsive gene was regulated in *BIL2*-*OX* and *BIL2*-*RNAi*

**Fig. 5:**
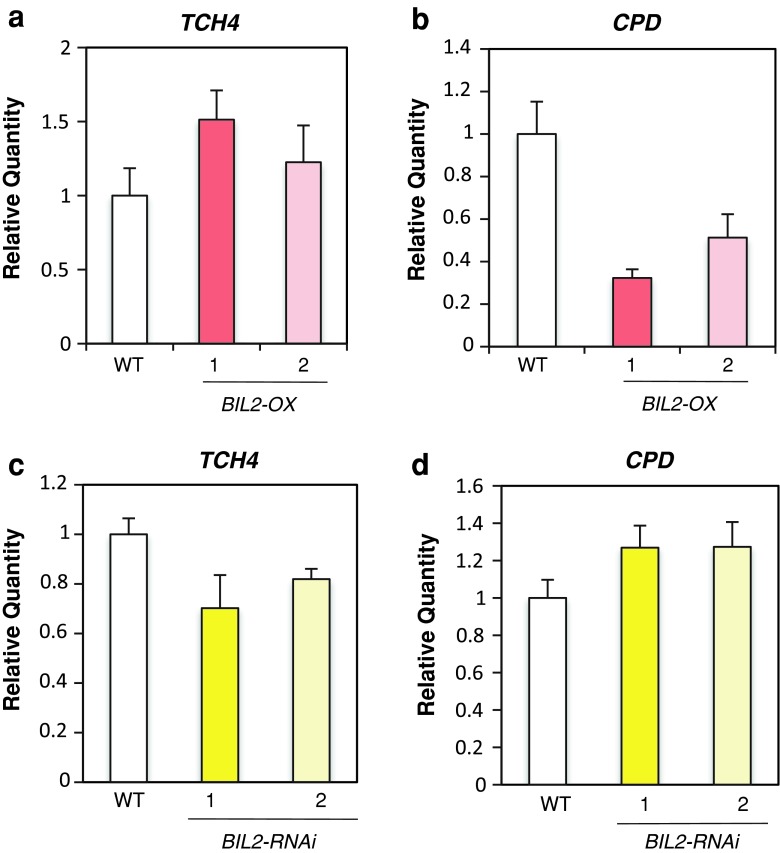
The BR-responsive gene was regulated in *BIL2*-*OX* and *BIL2*-*RNAi*. **a**, **b** Real-time PCR analysis of the regulated BR gene expression in the wild-type (WT), *BIL2*-*OX1* and *BIL2*-*OX2* seedlings grown in the light for 24 days. **c**, **d** Real-time PCR analysis of the regulated BR gene expression in the wild-type (WT), *BIL2*-*RNAi1* and *BIL2*-*RNAi2* seedlings grown in the light for 7 days. All values were normalized against the expression of the *ACTIN2* gene. The *error bars* indicate standard deviation (*n* = 3)

Although the hypocotyl insensitivity of *bil2*-*1D* and *BIL2*-*OX* against Brz showed that BIL2 protein can be related to BR signaling, the relationship between BIL2 and BR at the molecular level has not been clarified. To reveal the importance of BIL2 for BR signaling, BR-stimulated gene expression in *BIL2*-*OX* and *BIL2*-*RNAi* transformants was analyzed. Quantitative real-time PCR (qPCR) showed that the *BIL2* mRNA expression was higher in *BIL2*-*OX* (Fig. 3c) but lower in *BIL2*-*RNAi* compared with the wild-type plant (Fig. 4a). The BR-positive regulatory gene *THC4* showed higher expression, and the BR biosynthetic gene *CPD*, which is downregulated with BR stimulation through a feedback mechanism and showed lower expression in *BIL2*-*OX* than in the wild-type plant (Fig. 5a, b). In contrast, the BR-positive regulatory gene *TCH4* showed lower expression, and *CPD* showed higher expression in *BIL2*-*RNAi* compared with the wild-type plant (Fig. 5c, d). These results suggest that BIL2 plays an important role for BR signaling in the regulation of BR gene expression.

### BIL2 plays a role downstream of BRI1 in BR signaling

**Fig. 6:**
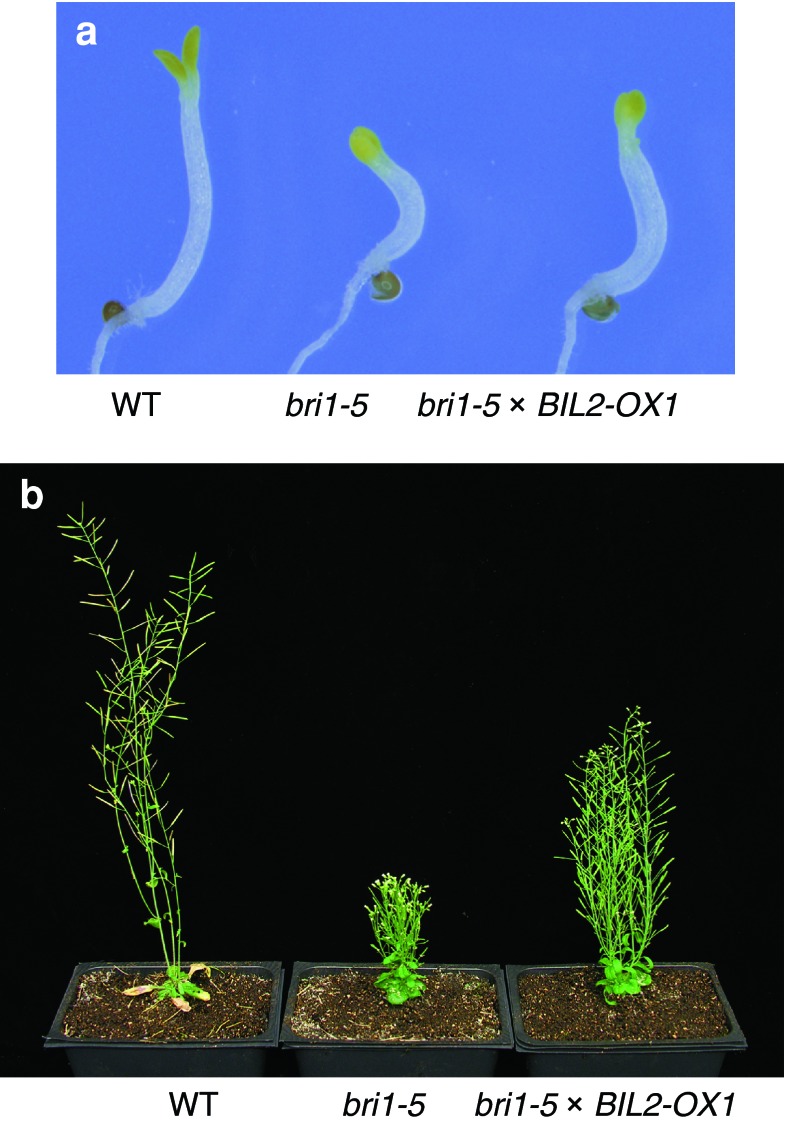
BIL2 plays a role downstream of BRI1 in BR signaling. **a**, **b** The *BIL2*-*OX* suppresses the dwarf phenotype of BR receptor mutant *bri1*-*5*, in the dark (**a**) and in the light (**b**). The plants of wild-type (WT), *bri1*-*5* and *bri1*-*5* × *BIL2*-*OX* seedlings grown on 3 μM Brz medium in the dark for 7 days (**a**) or in soil for 40 days (**b**)

BIL2 functions downstream of BR biosynthesis, as *bil2*-*1D* showed resistance against the BR biosynthesis inhibitor Brz (Fig. 1a, b), and functions upstream of BR gene expression (Fig. 5). To determine where BIL2 acts in BR signaling, the genetic interaction between *BIL2*-*OX* and the BR receptor mutant *bri1*-*5* was analyzed (Li and Chory 1997). When *BIL2*-*OX1* was crossed with *bri1*-*5*, *BIL2*-*OX1* suppressed shorter hypocotyl of *bri1*-*5* in the dark (Fig. 6a) and a shorter inflorescence of *bri1*-*5* in the light (Fig. 6b). These results suggested that the BIL2 plays an important role downstream of BRI1 in BR signaling.

### The *BIL2* gene is expressed in many plant organs

**Fig. 7:**
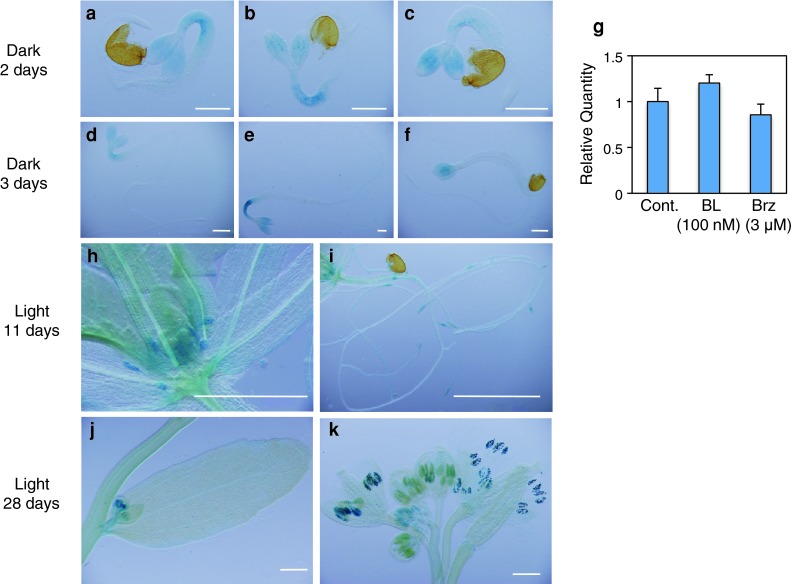
The *BIL2* gene is expressed in many plant organs. BIL2::promoter::GUS expression patterns in transgenic *Arabidopsis* plants. Dark 2- and 3-day-old seedlings on the 1/2 MS medium (**a**, **d**) treated with BL 100 nM (**b**, **e**) and 3 μM Brz (**c**, **f**). Real-time PCR analysis of BIL2 gene expression in wild-type seedlings grown in the light for 10 days and treated with 100 nM of BL and 3 μM of Brz (**g**). The values were normalized against expression of the *ACTIN2* gene. The *error bars* indicate the standard deviation (*n* = 3). Light-grown 11-day-old seedlings of the shoot apical meristem (SAM) (**h**) and root (**i**). Light-grown 28-day-old seedlings of flower bud (**j**) and pollen (**l**). *Scale bars* 0.5 mm (**a**–**f**, **h**) and 2 mm (**i**–**k**)

As *BIL2* is a novel gene, the analysis of *BIL2*-expressed organs can reveal the function of *BIL2* in plant growth. To investigate the specific expression of *BIL2* in various developmental stages of *Arabidopsis*, the *BIL2* promoter was constructed upstream of glucuronidase (GUS) and transformed into wild-type *Arabidopsis*. In 2 and 3 days after germination, *BIL2* was highly expressed in the hypocotyl after BL treatment (Fig. 7b, e), and low expression was observed in the hypocotyl after Brz treatment (Fig. 7c, f) in comparison with the hypocotyl in 1/2 MS medium (Fig. 7a, d). The qPCR analysis showed that the *BIL2* mRNA expression responses to BL and Brz were reproduced in the wild-type plants (Fig. 7g). The *BIL2* promoter region does not have a responsive element regulated by *BES1* and *BZR1*/*BIL1*; thus, an unknown functional element regulated by unknown transacting factors might exist in the *BIL2* promoter. At 11 days after germination, *BIL2* was highly expressed in the shoot apical meristem (SAM) and lateral root (Fig. 7h, i). At 28 days after germination, *BIL2* was expressed in the flowering bud and pollen (Fig. 7j, k). These results suggested that BIL2 plays an important role for plant development in many stages of the plant life cycle.

### The BIL2 protein localized in the mitochondria

**Fig. 8:**
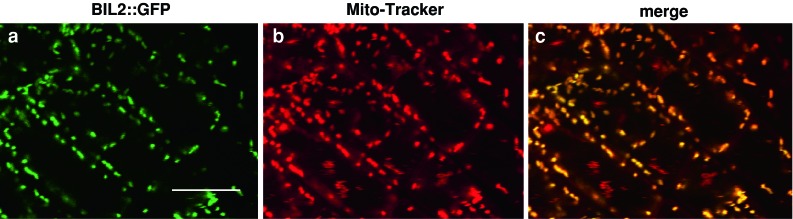
BIL2 protein localized in the mitochondria. Subcellular localization of BIL2 in transgenic plants. **a** *BIL2*-*GFP* localization in the root tip. **b** Mitotracker red dye fluorescence. **c** Merged image of **a** and **b**. *Scale bar* 10 μm

To determine the subcellular localization of the BIL2 protein, a *BIL2* promoter *BIL2* gene-green fluorescent protein (GFP) fusion construct was transformed into wild-type *Arabidopsis*. Observation by confocal scanning of the *BIL2*-*GFP* transgenic seedling roots revealed that the BIL2-GFP was observed in a dot-like structure (Fig. 8a). When co-stained with Mitotracker red, which specifically stains mitochondria (Hedtke et al. 1999) (Fig. 8b), the two staining patterns overlapped (Fig. 8c). The dot-like structure shown by *BIL2*-*GFP* did not co-localize with FM4-64 as an endosome marker, XylT-RFP as a Golgi marker and HDEL-RFP as an ER marker (Suppl. Fig. S3). These results indicated that BIL2 was localized to mitochondria.

### BIL2 may be involved in ATP synthesis in the mitochondria

**Fig. 9:**
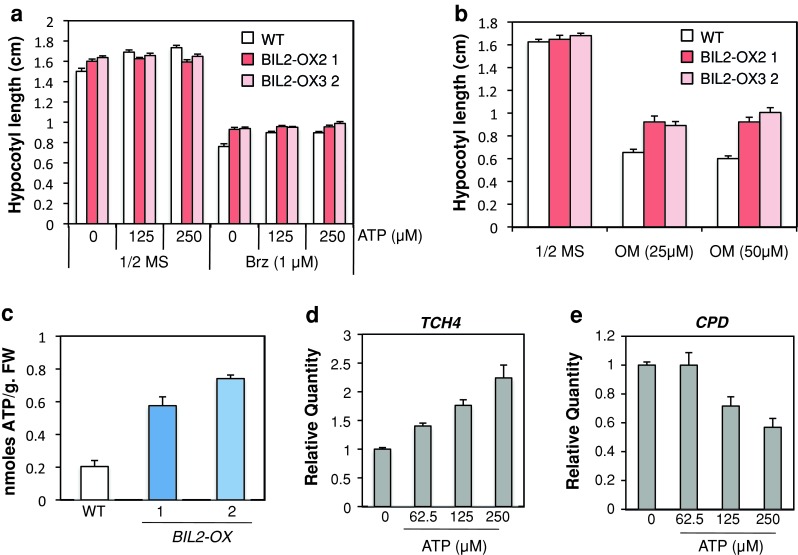
ATP production by BIL2 promotes hypocotyl elongation. **a** Hypocotyl elongation by ATP. Hypocotyl lengths of dark 7-day-old seedlings grown in the absence or presence of 1 μM Brz or ATP (125 and 250 μM). Combinatorial treatments are indicated. **b** Hypocotyl lengths of dark 7-day-old seedlings grown in the absence or presence of oligomycin (OM) (25 and 50 μM). Twenty-five seedlings per treatment were analyzed in each experiment. The data shown are the mean ± SE. **c** Total ATP concentration in dark-grown 5-day-old seedlings of wild-type (WT), *BIL2*-*OX1* and *BIL2*-*OX2*. The data shown are the mean ± SE of three independent experiments. **d**, **e** ATP can increase BR signaling. Real-time PCR analysis of regulated BR gene expression in wild-type (WT) seedlings grown in the dark for 7 days and treated with ATP for 3 h

Although the BR signaling factor localized in many plant organelles (Belkhadir et al. 2006), the BR signaling factor in mitochondria had not been determined. To consider the molecular mechanism of plant growth regulation in BR signaling by the mitochondrial protein BIL2, we noted that ATP synthesis that was a major role of mitochondria for plant growth. The relationship between BIL2 and ATP in the hypocotyl elongation was then analyzed. Exogenous ATP promotes hypocotyl elongation and also restores hypocotyl elongation, which was shortened by Brz treatment in the dark (Fig. 9a). The hypocotyl elongation of *Arabidopsis* is inhibited by oligomycin, an inhibitor of the respiratory chain complex V of the mitochondrial electron transport and a blocker of H+-ATP synthesis. The hypocotyl of *BIL2*-*OX* showed partial but significant resistance against oligomycin-induced inhibition of hypocotyl elongation in comparison with the wild-type plant (Fig. 9b). To reveal the direct interaction between ATP synthesis and BIL2, the ATP concentration in the *BIL2*-*OX* and wild-type plant was analyzed. In dark-germinated plants for 5 days, the endogenous ATP in two lines of *BIL2*-*OX* was higher than in the wild-type plant (Fig. 9c). To analyze the relationship between ATP and BR signaling in *Arabidopsis*, BR-responsive gene expression was analyzed in the *Arabidopsis* wild-type plants after a 3-h ATP treatment (Fig. 9). In the plants germinated in the dark for 7 days, *TCH4* expression, which is upregulated by BR stimulation, showed increasing dependence on the ATP concentration (Fig. 9d). By contrast, the BR biosynthetic gene *CPD*, the expression of which is downregulated by BR stimulation through a feedback mechanism, showed decreasing expression that was dependent on the ATP concentration (Fig. 9e). These results suggested that ATP synthesis was promoted through the action of BIL2 in the mitochondria and elongated the hypocotyl against the BR biosynthesis inhibitor Brz. BR signaling-related gene expression was also promoted by ATP synthesis through BIL2.

### BIL2 increases salt tolerance and strong light tolerance in *Arabidopsis*

**Fig. 10:**
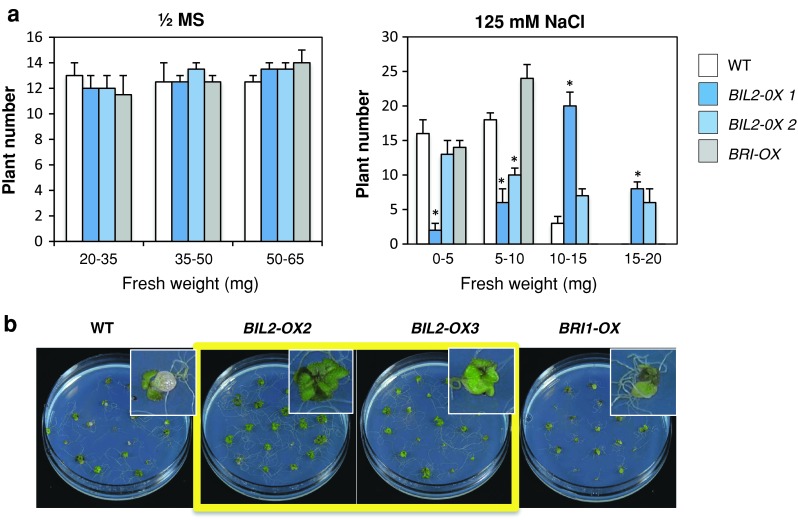
*BIL2*-*OX* showed resistance against salt stress. **a** Fresh weight above-ground parts of each plants were divided in four groups (0–5, 5–10, 10–15, and 15–20 mg) and the plant numbers were counted in each group. Individual numbers of the wild-type (WT), *BIL2*-*OX1*, *BIL2*-*OX2* and *BRI*-*OX* seedlings germinated on half-strength MS medium with (*right graphics*) or without (*left graphics*) 125 mM NaCl and under light for 25 days. **b** *BIL2*-*OX* and wild-type (WT) plant phenotypes under salt stress. Photographs of the seedlings were obtained at 25 days after germination. The data shown are the mean ± SE of three independent experiments. *Asterisk* indicates significant differences relative to the control at *P* < 0.05 based on Student’s *t* test

**Fig. 11:**
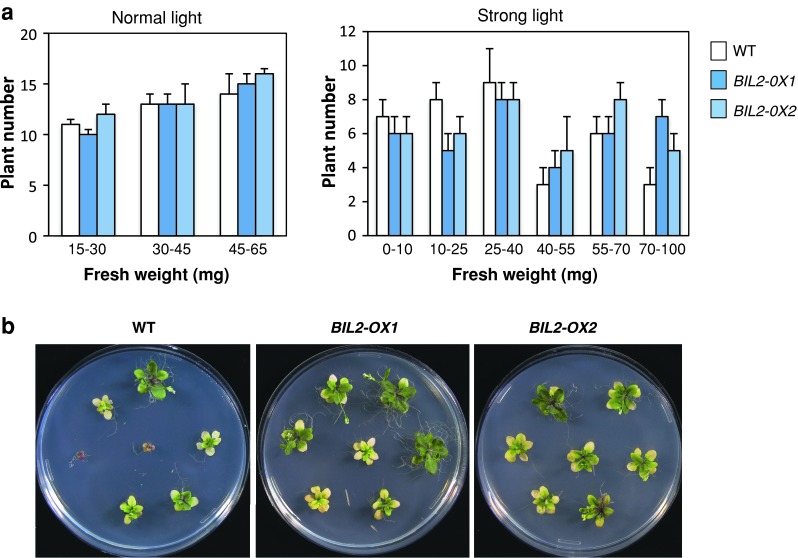
*BIL2*-*OX* showed resistance against strong light stress. **a** The number of plants was counted using the same method as with the salt resistance analysis explained in Fig. 12. The individual numbers of the wild-type (WT), *BIL2*-*OX1* and *BIL2*-*OX2* seedlings germinated on half-strength MS medium under normal light (*left graphics*) or strong light (*left graphics*) for 25 days. **b** *BIL2*-*OX* and wild-type (WT) plant phenotype in strong light stress. Photographs of the seedlings were obtained at 25 days after germination. The data shown are the mean values

Although the *BIL2* gene is a novel gene, it has been classified as a DnaJ gene family member in previous reports (Rajan and D’Silva 2009). The DnaJ gene family is included in heat shock proteins (HSPs) that play roles to protect against proteotoxic stress and to regulate the protein quality control system for assistance (Vos et al. 2008). To reveal the possible molecular function of BIL2, the environmental stress tolerance of the wild-type plant and *BIL2*-*OX* was analyzed. For the analysis of salt tolerance, plants grown on medium were divided into groups according to the fresh weight of the above-ground parts in plants grown in 1/2 MS medium and NaCl medium, and the numbers of each group were counted. The number of heavy and surviving plants of *BIL2*-*OX* grown on 125 mM NaCl was higher than wild-type plants (Fig. 10a, b). In the analysis of strong light stress tolerance, the number of heavy and surviving plants of *BIL2*-*OX* grown in the strong light (486.2 μmol m^−2^ s^−1^) was higher than wild-type plants (Fig. 11a, b). The reaction of *BRI1*-*OX* was similar to that of wild-type plants under salt stress (Fig. 10) and strong light stress (data not shown). These results suggest that *BIL2*-*OX* shows potential resistance against salt and strong light stress that depends on the overexpression of *BIL2*. Thus, BIL2 maintains its function to protect against proteotoxic stress and can be classified as a DnaJ family protein according to protein function. ATP treatment promoted salt tolerance in the wild-type plants (Suppl. Fig. S4), and the direct treatment of BL caused a weak salt tolerance in the wild-type plants (data not shown). These results showed that although ATP synthesis supported by BIL2 plays important roles, the direct BL signaling effect might be weaker than ATP itself.

## Discussion

BR regulates many processes in plant growth and development, such as cell division and elongation (Clouse and Sasse 1998). BR biosynthesis-deficient mutants of *Arabidopsis*, *de*-*etiolated 2* (*det2*) and *dwrf4*, (Li et al. 1996; Choe et al. 1998) showed a pleiotropic dwarf phenotype that can be recovered to a wild-type phenotype by feeding of BR (Asami et al. 2005); however, the BR receptor-deficient mutant *bri1* displays a pleiotropic dwarf phenotype, including root elongation, which was not rescued by BR (Clouse et al. 1996). BR binds with BRI1, a member of the leucine-rich repeat kinase family (Li and Chory 1997; Kinoshita et al. 2005). The detailed mechanism downstream of BRI1 in BR signaling has been studied, and all these results have been revealed through loss-of-function mutants for the biosynthesis of BR and the BR receptor. BIN2, BZR1/BIL1 and BES1, the other BR signaling components, were identified through gain-of-function mutants.

Brz is a specific inhibitor of BR biosynthesis that inhibits the hydroxylation of the C-22 position of the side chain in BR by direct binding to DWF4 enzyme, a cytochrome P450 monooxygenase, through the triazole base of Brz (Asami et al. 2001). To analyze the mechanisms of BR signal transduction, we performed a chemical genetics screening using Brz from gain-of-function mutants. We screened *Arabidopsis* activation tagging lines and isolated the *bil2*-*1D* mutant, which displayed longer hypocotyls characteristic of cell elongation on medium containing Brz in the dark than the wild-type plant. Light-grown *bil2*-*1D* exhibited a long petiole phenotype similar to wild-type plants treated with BR or *BRI1*-*OX* mutants. The BR marker gene *TCH4*, the expression of which was upregulated by BR stimulation, was induced in the *BIL2*-*OX* but not in the wild-type. Conversely, *CPD*, the other BR biosynthetic gene, the expression of which is downregulated by BR stimulation, was suppressed in the *BIL2*-*OX* but not in the wild-type. *BIL2*-*OX* could suppress dwarfing of the *BRI1*-deficient mutant. *BIL2* gene expression was induced by BL treatment and suppressed by Brz, suggesting that *BIL2* is directly regulated by BL and is involved in cell elongation through BR signaling.

BIL2 is a novel protein, but analysis of the amino acid sequence revealed that it belongs to the DnaJ/Hsp40 family proteins. DnaJ/Hsp40 proteins are functional partners for DnaK/Heat shock protein 70 (Hsp70s) involved in protein folding, translation, stabilization and protein translocation across cell membrane. All members of DnaJ/Hsp40 contain a “J domain”, which is essential for interaction with DnaK/Hsp70s. The J domain contains a highly conserved histidine, proline and aspartate (HPD) motif, which is critical for their functions. Some members of the DnaJ/Hsp40 protein family contain other conserved regions, such as the glycine/phenylalanine rich region, termed the “G/F region” and a zinc-binding cysteine-rich sequence, termed the “zinc-finger domain”. DnaJ/Hsp40 proteins are classified into three types on the basis of differences in these regions (Szyperski et al. 1994; Cheetham and Caplan 1998). Type I proteins contain all domains/motifs that include the J domain, the G/F region, and the zinc-finger domain. Type II proteins possess the J domain and the G/F region, but lack the zinc-finger domain. Type III proteins possess only the J domain (Kelley 1998; Fan et al. 2004; Walsh et al. 2004). BIL2 is classed as a type III J protein (Rajan and D’Silva 2009). DnaJ/Hsp40 is widely distributed in plants, animals and humans. The DnaJ/Hsp40 protein family is composed of six homologs in *Escherichia coli*, 22 homologs in *Saccharomyces cerevisiae* and 41 homologs in humans (Qiu et al. 2006). *Arabidopsis* has more than 400 DnaJ/Hsp40 protein families (Rajan and D’Silva 2009). These family protein functions have not been elucidated, and the BIL2 functions were not known. The J domain amino acid sequence of BIL2 is similar to the human DnaJ protein that possesses a tetratricopeptide repeat 2 (TPR2). The TPR proteins function in various cellular processes, including cell-cycle control, mitochondrial and peroxisomal protein transport, stress response, and protein kinase inhibition (Goebl and Yanagida 1991). DnaJ/Hsp40 proteins regulate DnaK/Hsp70 and other proteins as a function of chaperones. The DnaJ/Hsp40 protein TPR2 (DnaJC7) is involved in the folding of many proteins, and TPR2 mediates the retrograde transfer of substrates from Hsp90 to Hsp70 (Brychzy et al. 2003).

*BIL2*-*GUS* expression was observed in hypocotyl during early stage and strongly expressed in pollen during in the flower developmental stage. BR biosynthetic and signaling deficient mutants showed reduced pollen number, viability, and release efficiency (Ye et al. 2010). *MALE GAMETOPHYTE DEFECTIVE 1* (*MGP1*) encodes the F_A_d subunit of mitochondrial F_1_F_0_-ATP synthase in *Arabidopsis* which was highly expressed in pollen and plays important roles in pollen formation (Li et al. 2010).

BIL2 localization was detected in the mitochondria. Although BR signaling proteins are localized in many organelles, i.e., cellular membrane, nuclei, endoplasmic reticulum (ER) and vacuole (Belkhadir et al. 2006), this study is the first to report BR signaling protein localization in the mitochondria. Our most interesting but difficult discovery during the BIL2 analysis was the localization of BIL2 in mitochondria. We would like to discuss regulation mechanism of BR signaling and plant growth by the mitochondrial protein.

**Fig. 12:**
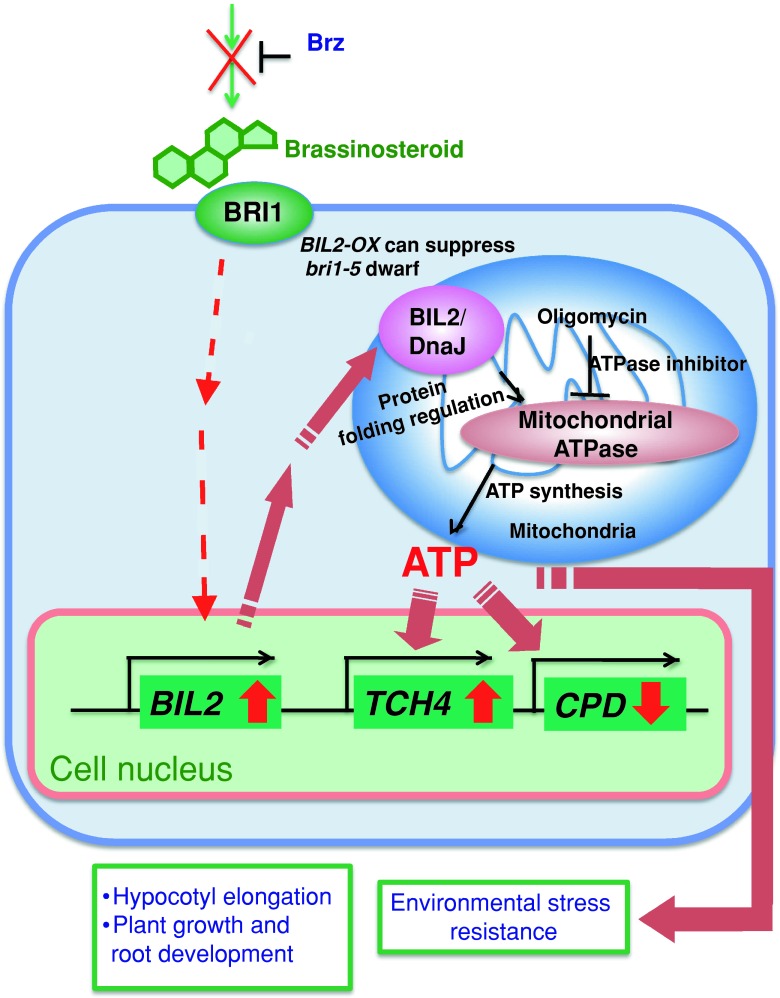
Schematic representation of BIL2 in the mitochondria. BIL2 induces cell elongation through BR signaling to promote ATP synthesis in the mitochondria

ATP is a vital factor in plant growth, essentially representing a major energy source of the cell. Most of the plant ATP is primarily produced in the mitochondria and secondarily in the chloroplast (Haferkamp et al. 2011). Therefore, understanding the mechanisms involved in ATP synthesis in the mitochondria is important. We hypothesized that ATP synthesis in the mitochondria by ATPase promotes hypocotyl elongation, and ATPase folding or stabilization might be facilitated through BIL2 as a DnaJ/Hsp40 function in the mitochondria. Although wild-type seedlings grown in medium containing Brz had shorter hypocotyls, *BIL2*-*OX* had a longer hypocotyl due to its resistance to Brz. The wild-type hypocotyl phenotype was recovered after treatment with ATP in medium containing Brz. These results showed that ATP plays an important role in hypocotyl elongation against Brz. The mitochondrial ATPase inhibitor oligomycin, which blocks the respiratory chain complex, inhibited hypocotyl elongation, but hypocotyl elongation of *BIL2*-*OX* was resistant to oligomycin compared to the wild-type plant. *BIL2*-*OX* produced higher exogenous ATP than the wild-type plant. These results support our hypothesis that BIL2 facilitates the folding or stability of ATPase in the mitochondria during plant growth (Fig. 12).

Environmental stress causes misfolding, aggregation and degradation for each organelle protein in the plant. In plant mitochondria, these proteotoxic damages have been observed, and ATP generation plays an important role in stress resistance (Jacoby et al. 2011). *BIL2*-*OX* showed resistance against salinity stress and strong light stress. ATP treatment promoted salinity resistance in wild-type *Arabidopsis* grown in medium containing NaCl (Suppl. Fig. S4). BIL2 is classified as a member of the DnaJ/Hsp40 family (Rajan and D’Silva 2009), but the actual function of the BIL2 protein has not been elucidated. The effects of BIL2 resistance against environmental stress might support the function of BIL2 as a DnaJ/Hsp40 molecular chaperone in the plant mitochondria. Further analysis will reveal the function of BIL2 in detail.

## Electronic supplementary material

Below is the link to the electronic supplementary material.

Supplementary material 1 (TIFF 2926 kb)

Supplementary material 2 (TIFF 2926 kb)

Supplementary material 3 (TIFF 2926 kb)

Supplementary material 4 (TIFF 2926 kb)

## Abbreviations

BIL2: Brz-insensitive-long hypocotyls 2
BIL2-OX: *BIL2* overexpression
BRI1: BRASSINOSTEROID INSENSITIVE 1
BKI1: BRI1 KINASE INHIBITOR 1
BAK1: BRI1-ASSOCIATED RECEPTOR KINASE 1
BSU1: *bri1*-*5* suppressor 1
BIN2: BRASSINOSTREROID INSENSITIVE 2
BZR1: BRASSINOZOLE RESISTANT 1
BES1: *bri1*-EMS-SUPPRESSOR 1
BR: Brassinosteroid
BL: Brassinolide
Brz: Brz220
CaMV: Cauliflower mosaic virus
DnaJ: J-protein, Hsp40
Hsp40: 40 kDa heat shock protein
HPD motif: Histidine, proline, aspartate motif
MS: Murashige and Skoog
RNAi: RNA interference
TAIL-PCR: Thermal asymmetric interlaced PCR
