# Supplementary figures and images for: A novel mitochondrial DnaJ/Hsp40 family protein BIL2 promotes plant growth and resistance against environmental stress in brassinosteroid signaling
Source: Planta. 2013 Mar 15;237(6):1509–25. doi: 10.1007/s00425-013-1859-3 (PMC3664749; doi:10.1007/s00425-013-1859-3)

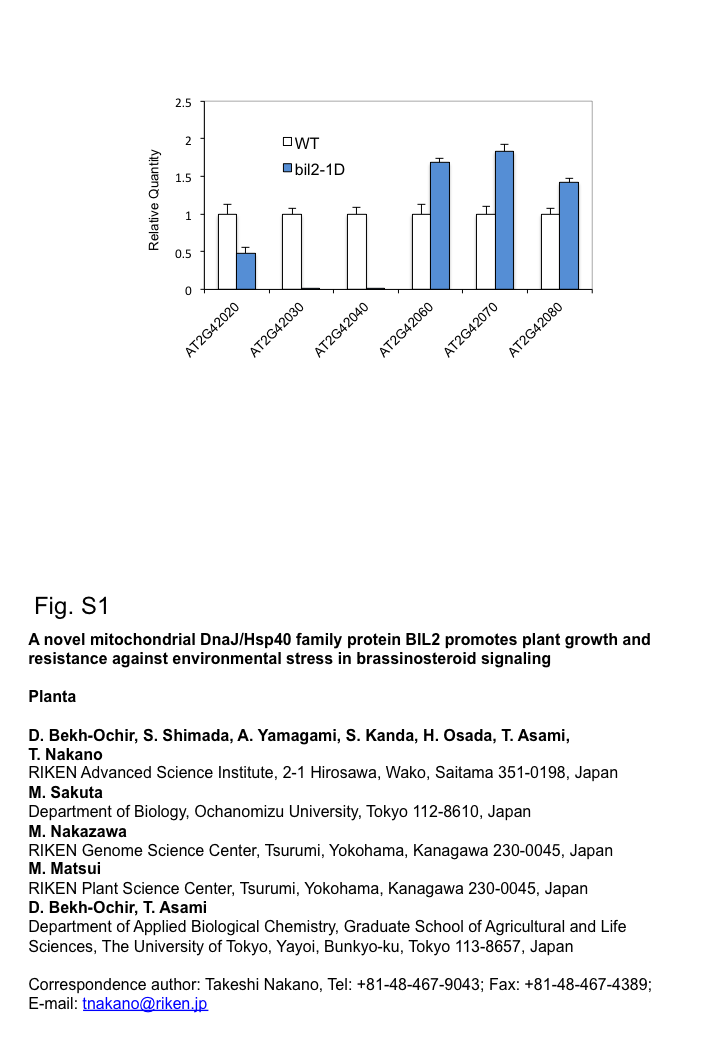

Supplement: Supplementary file 1 — Supplementary material 1 (TIFF 2926 kb) [file 425_2013_1859_MOESM1_ESM.tiff]

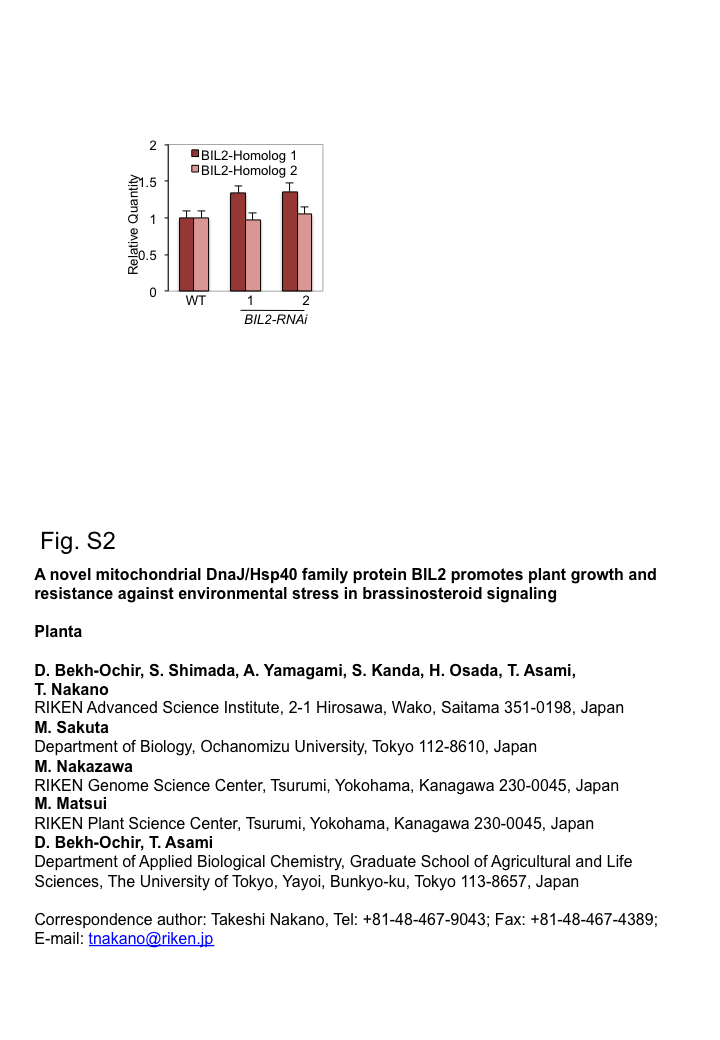

Supplement: Supplementary file 2 — Supplementary material 2 (TIFF 2926 kb) [file 425_2013_1859_MOESM2_ESM.tiff]

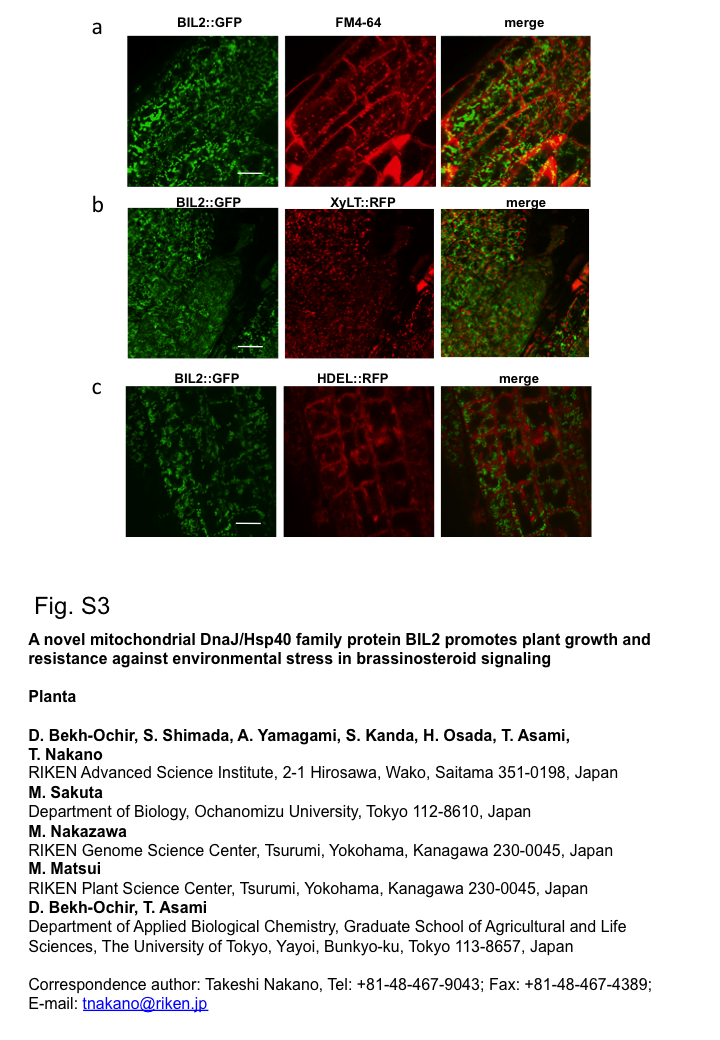

Supplement: Supplementary file 3 — Supplementary material 3 (TIFF 2926 kb) [file 425_2013_1859_MOESM3_ESM.tiff]

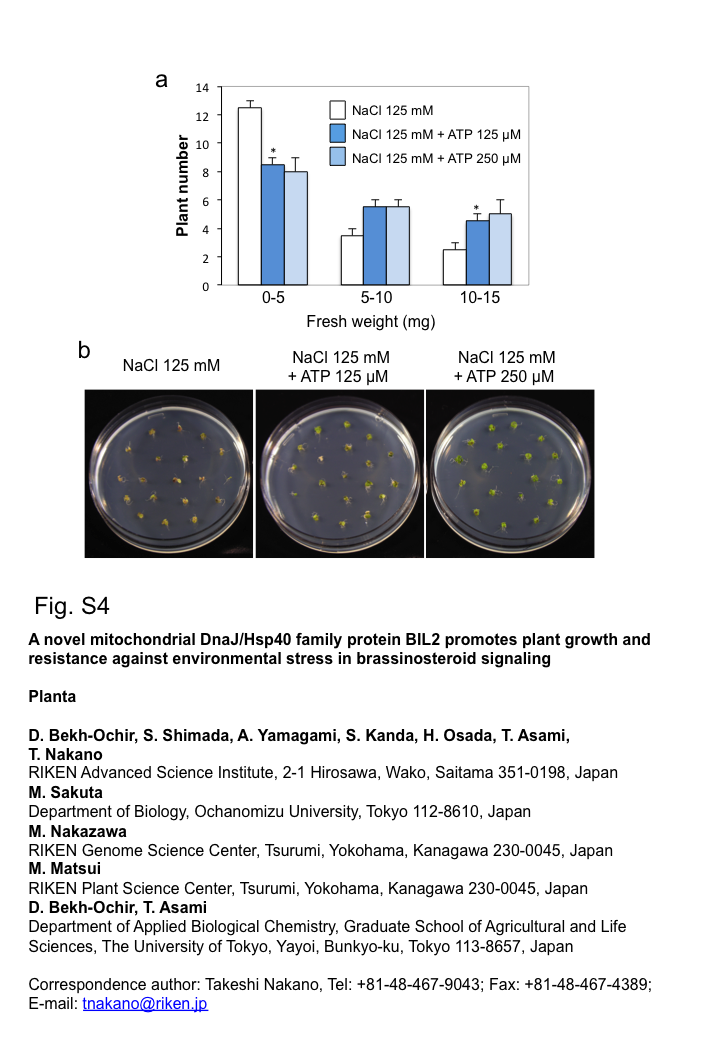

Supplement: Supplementary file 4 — Supplementary material 4 (TIFF 2926 kb) [file 425_2013_1859_MOESM4_ESM.tiff]
